# Hospital-acquired invasive pulmonary aspergillosis in patients with hepatic failure

**DOI:** 10.1186/1471-230X-8-32

**Published:** 2008-07-31

**Authors:** Dan Li, Liang Chen, Xian Ding, Ran Tao, Yong Xin Zhang, Jie Fei Wang

**Affiliations:** 1 Shanghai Public Health Clinical Center affiliated to Fudan University, Shanghai, 210508, PR China; 2 Huashan Hospital affiliated to Fudan University, Shanghai, 200040, PR China

## Abstract

**Background:**

Invasive pulmonary aspergillosis (IPA) is a rapid, progressive, fatal disease that occurs mostly in immunocompromised patients. Patients with severe liver disease are at a heightened risk for infections. Little is known about the clinical presentation including predisposing factors and treatment of IPA in patients with hepatic failure.

**Methods:**

Medical records of patients with hepatic failure between November 2005 and February 2007 were reviewed for lung infection. Nine medical records of definitive diagnosis of IPA and three of probable IPA were identified.

**Results:**

The main predisposing factors were found to be prolonged antibiotic therapy and steroid exposure. Clinical signs and radiological findings were non-specific and atypical. Timely use of caspofungin was found to reduce the mortality due to the disease.

**Conclusion:**

A high index of suspicion is required for early IPA diagnosis in patients with hepatic failure.

## Background

*Aspergillus *is a saprophytic filamentous fungus widespread in the environment, and has attracted attention in recent years for its association with various clinical conditions, depending essentially on the host's immunological status [1,2]. In immunocompetent patients, pulmonary aspergilloma, allergic aspergillus tracheobronchitis and obstructive bronchial aspergillosis are described. In immunocompromised patients, it can invade the pulmonary parenchyma, resulting in invasive pulmonary aspergillosis (IPA), a disease with high lethality [1-4].

Patients with severe liver disease are at a higher risk for various infections [5,6]. Little is known about the clinical presentation and treatment of IPA in patients with hepatic failure. The available literature is confined to a few case reports or series of reports only, with data mainly based on autopsy reports. The aim of the present study was to determine the clinical presentation and outcome of aspergillosis in patients with hepatic failure. In contrast to other reports, only one out of 12 patients diagnosed with IPA died. We have analyzed predisposing factors and treatment regimen, in an effort to determine the basis for low mortality in our patient cohort.

## Methods

This was a retrospective study, conducted in Shanghai Public Health Clinical Center, a tertiary care hospital specializing in infectious diseases, especially hepatic diseases. Between November 2005 and February 2007, a total of 276 patients with hepatic failure were admitted. Medical and microbiological records as well as the chest radiographic imaging of all patients with hepatic failure and accompanying lung infection were reviewed. Patients' demography, duration of admission, predisposing factors, clinical features, investigations, treatment and outcome were noted.

Standardized criteria from European Organization for Research and Treatment of Cancer and the Mycosis Study Group of the National Institute of Allergy and Infectious Disease (EORTC/MSG) [7] were applied for the diagnosis of definite and probable IPA. Definite IPA was defined as the demonstration of filamentous fungi by microscopy from tissue samples with or without a positive culture for *Aspergillus*. Probable IPA was defined as the demonstration of filamentous fungi compatible with the morphology of *Aspergillus *and/or a positive culture for *Aspergillus *from bronchoalveolar lavage (BAL) specimen in patients in conjunction with 1 major (halo or "air crescent" on computed tomography (CT) scan) or at least two minor (signs of lower respiratory tract infection, pleural rub, and presence of any new infiltrate in a patient who did not fulfill the major criterion but for whom no alternative diagnosis was available) clinical findings. IPA developing after 48 hours of hospital admission and before the clinical diagnosis of fungal infection was considered nosocomial; otherwise, the fungal infection was regarded as community acquired.

Serological diagnosis, such as galactomannan or β-d-glucan, are not performed in Shanghai Public Health Clinical Center; thoracic CT imaging combined with the patient's condition despite antibiotic treatment was the main approach for preliminary diagnosis of IPA. In brief, appearance of pulmonary consolidation or infiltrate and rapid progression on thoracic CT scan with antibiotic resistant fever in the appropriate host setting was diagnosed as suspected IPA. Single-bed hospital room was provided for the patient with high index of suspicion of IPA. In patients with pulmonary infiltrates, especially those with diffuse pulmonary infiltrates, fiberoptic bronchoscopy with BAL and/or transbronchial lung biopsy (TBLB) was performed. In patients with focal pulmonary lesions, percutaneous puncture lung biopsy (PPLB) was considered the first-line diagnostic tool. BAL or lung biopsy was performed at the request of the treating physician with informed consent.

Thoracic CT scan was performed early in patients suspected of having IPA, and twice a week for early detection of IPA or preliminary evaluation of antifungal therapy. Results of chest X-ray and thoracic CT scan were described as normal, grand gross attenuation, non-specific infiltrates and consolidation, pleural fluid, nodular lesion(s), halo sign, air-crescent sign, and cavitation. The CT halo sign is described as a surrounding halo of ground glass attenuation surrounding a pulmonary nodule or mass and corresponds to a central fungal nodule surrounded by a rim of hemorrhage and coagulative necrosis. The air-crescent sign is described as a pulmonary cavitation [8].

All continuous data were expressed either as mean and standard deviation (SD), or median and range based on the distribution.

The study was approved by the Research Ethics Committee, Shanghai Public Health Clinical Center, Fudan University.

## Results

Examination of the medical records of all 276 patients admitted to Shanghai Public Health Clinical Center with hepatic failure identified 18 patients who had suspected IPA with a negative sputum culture; of these, seven had suspected IPA on admission. Chest CT showed that all patients had a two fold increase in lesion volume within the first three days despite broad spectrum antibiotic treatment. Among the 18 patients, one stopped treatment due to economic constraints; two objected to invasive examination but agreed to antifungal therapy. Six patients underwent BAL; of these, two patients also had TBLB. Lung biopsy was carried out in 12 patients, of which eight were TBLB and four were PPLB. Five patients had a positive BAL culture for *Aspergillus*, but biopsy did not show any abnormalities in the tracheobronchial tree. Nine of twelve biopsies were histologically positive for *Aspergillus*, the others were positive for *Enterococcus faecium, Cytomegalovirus *and *Mucor*, respectively. Thus, a total of 12 patients were involved in this study, with definitive diagnosis of IPA in 9 and probable IPA in 3 patients. The median time to diagnosis was 8 days (range 1–22 days) after admission. Among the patients who underwent biopsy, nine had no complications, one case had pneumothorax with lung compressed to less than 20% and 2 cases presented with hemoptysis.

All 12 were referral cases; five of them were transferred for deteriorated pneumonia from local hospitals and suspected of IPA at admission. The initial status of all 12 patients at admission is listed in Table 1. The cause of hepatic failure was hepatitis B virus (HBV) infection in eleven patients, and coinfection with HBV and hepatitis delta virus (HDV) in one patient. IPA was nosocomially acquired in all patients, but none of these infections were acquired in the intensive care unit.

**Table 1:** Demographic features of 12 patients with IPA

Characteristic	Number of patients
Age in years (mean ± S.D.)	41.9 ± 9.5
Males (%)	87.5
Disease onset	
Acute hepatic failure	0
Subacute hepatic failure	3
Chronic hepatic failure	9
Complications	
Hepatic encephalopathy	5
Hepatorenal syndrome	3
Spontaneous bacterial peritonitis	3
MELD score (median, range)	30 (22–51)
Antibiotic usage	
Duration (days, mean ± S.D.)	23.1 ± 8.6
One antibiotic	5
Two antibiotics	4
Three or more antibiotics	3
Steroid exposure	9

The chief symptoms were mild to moderate fever and decrease in mental function. Eight patients (66.7%) did not have any respiratory symptoms (Table 2). All had non-specific radiological findings. The halo and air-crescent sign were evident in only two patients (Table 2). CT brain scan was performed in six patients who had psychiatric symptoms; one patient was found to have a mass-like low density lesion with a surrounding halo in the right temporal lobe and clinically diagnosed as having encephalic aspergillosis. Clinical features of IPA patients before antifungal treatment are summarized in Table 3.

**Table 2:** Clinical and laboratory parameters of patients with IPA

Signs or symptoms	Number of patients
Fever	12
Cough	3
Chest pain	0
Hemoptysis	0
Crackles	1
*Chest X-ray findings*	
Changes in bilateral lung fields	9
Bilateral lung fields diffused	2
Right unilateral lung	2
Left upper lobe	4
Halo and air-crescent	2
*Laboratory findings*	
Hemoglobin (g/L, mean ± S.D.)	95.7 ± 15.9
Leukocyte counts (×10 ^9^/L, mean ± S.D.)	11.6 ± 7.9
Neutrophils (%, mean ± S.D.)	81.2 ± 6.4
Thrombocyte count (×10^9^/L, mean ± S.D.)	102.7 ± 69.5
Total bilirubin (μmol/L, mean ± S.D.)	661.3 ± 189.7
Prothrombin activity (%, range)	8–26
HBV viral load (copies/ml, range)	10^4^–10^7^

**Table 3:** Clinical features of patients with confirmed IPA

Age/gender	Cause of HF	MELD score	Complications of HF	Co-morbid conditions	Outcome
32/M	HBV	37.12	HE	PE	Recovered
44/M	HBV	29.16	HRS	PE	Recovered
38/M	HBV	31.78	HE	AP	Recovered
51/F	HBV	32.66		PE	Recovered but paralyzed
64/M	HBV, HDV	35.01	HRS	PE	Recovered
47/M	HBV		HE		Recovered
39/M	HBV	38.97	SBP, HRS, HE		Dead
52/M	HBV	33.14		DM	Recovered
41/M	HBV	30.63		PE	Recovered

MELD: model for end liver disease; PE: plasma exchange; HF: hepatic failure; HBV: hepatitis B virus; HDV: hepatitis D virus; HE: hepatic encephalopathy; HRS: hepatorenal syndrome; DM: diabetes mellitus; SBP: spontaneous bacterial peritonitis; AP: acute pancreatitis.

All patients received antibiotic therapy and nine patients had steroid exposure before IPA diagnosis (Table 1). Steroids were used as prevention for allergic reactions to plasma, or to decrease persistent hyperbilirubinemia. The maximum amount of steroids administrated was 1.8 gram of methylprednisolone in one patient.

Patients were hospitalized for 15 to 221 days. Six patients were in intensive care unit for ten or more days with APECHE II score range from 17–29, four required noninvasive assisted ventilation, and one died two days after definite diagnosis.

The median time from suspicion of fungal infection to antifungal treatment was 4 days (range from 1 ~ 16 days). All patients (except the one patient who died) were administrated caspofungin as antifungal agent after diagnoses for at least 3 weeks (3–12 weeks), the patient with endocranial aspergillosis received additional voriconazole for six months. Cessation of antifungal treatment is subjectively judged by physicians based on patient's improved condition, disappearance of clinical symptoms, and improved laboratory tests. CT scan imaging was not used as the evaluation measure, as four patients still had small lamellar high density lesion on CT imaging. After a median follow-up of 7 months (range from 4 to 11 months), all surviving patients were well, the residual lesion on CT scan disappeared in three cases, with significant decrease in a fourth patient.

## Discussion

The development of IPA is almost always observed in immunosuppressed individuals with an impairment or deficiency of phagocyte function [9]. It has been previously demonstrated that decompensated liver disease is an acquired immune deficiency state [5,6]; dysmetabolism and malnutrition are also contributing factors. Patients with severe liver disease are more prone to various infections. Patients in our study presented two risk factors for invasive pulmonary aspergillosis, broad spectrum antibiotic usage and steroid exposure, according to the criteria defined by the EORTC/MSG [7]. These elements lend to the notion that narrow-spectrum antibiotic is preferred and long term antibiotic treatment should be avoided; corticosteroids should be used with caution in patients with hepatic failure.

Most patients in this study had atypical clinical presentation often disregarded by physicians due to underlying hepatic dysfunction and hepatic encephalopathy, and radiological features were no different from bacterial pneumonias. It should be noted that nine of twelve (75%) biopsies performed on patients with suspected IPA confirmed this diagnosis. This rate was achieved because a high index of suspicion exists for the occurrence of invasive pulmonary aspergillosis in patients with hepatic failure. An aggravated condition during the usage of antibiotics, such as an unexpected breakdown in mental status, a marked increase in total serum bilirubin, a decrease in PTA with antibiotic resistant fever, indicates suspected fungal infection [10]. Although *Candida monilia *is the most common pathogen in clinical fungal infection, the change in leukocyte counts and percentage of neutrophils may be used for differential diagnosis of *Candida monilia *and *Eurotium *infection [11]. A rapid progression on thoracic CT scan with no response to antibiotic therapy would reinforce the judgment [8]. In our study, the leukocyte counts were elevated in 91.7 (11/12) patients, with increased percentage of neutrophils in all patients.

In our study, caspofungin dramatically reversed the high mortality of the disease reported by others [12,13], while therapy to rebuild the immune system was not adopted, as hepatic injury caused by HBV uses immunological mechanisms.

## Conclusion

Atypical clinical presentation of IPA was found in 12 patients with hepatic failure, suggesting that a high index of suspicion is required for timely diagnosis of IPA. Early and effective anti-fungal treatment greatly improves the prognosis.

## Competing interests

The authors declare that they have no competing interests.

## Authors' contributions

All authors contributed to the design of the study. Three authors (LC, XD and RT) collected and analyzed the data, the others made interpretation and final decision when questions arised, two authors (DL and LC) have been involved in drafting the manuscript.

## Pre-publication history

The pre-publication history for this paper can be accessed here:


